# Methylphenidate Treatment of Cognitive Dysfunction in Adults After Mild to Moderate Traumatic Brain Injury: Rationale, Efficacy, and Neural Mechanisms

**DOI:** 10.3389/fneur.2019.00925

**Published:** 2019-09-12

**Authors:** Harvey Levin, Maya Troyanskaya, JoAnn Petrie, Elisabeth A. Wilde, Jill V. Hunter, Tracy J. Abildskov, Randall S. Scheibel

**Affiliations:** ^1^Department of Physical Medicine & Rehabilitation, Baylor College of Medicine, Houston, TX, United States; ^2^Michael E. DeBakey Veterans Affairs Medical Center, Houston, TX, United States; ^3^Department of Neurology, University of Utah, Salt Lake City, UT, United States; ^4^George E. Wahlen VA Salt Lake City Healthcare System, Salt Lake City, UT, United States; ^5^Baylor College of Medicine, Texas Children's Hospital, Houston, TX, United States

**Keywords:** traumatic brain injury, methylphenidate, clinical trials, imaging, dopamine, cognition

## Abstract

Positive effects of methylphenidate (MPH) on attention and cognitive processing speed have been reported in studies of patients with moderate to severe traumatic brain injury (TBI). Studies which have acquired functional brain imaging before and while using MPH have also found alteration of brain activation while performing a cognitive task; in some studies, this alteration of activation in selective brain regions was also related to improved performance on cognitive tests administered outside of the scanning environment. Enhanced cognitive performance has been reported after single doses of MPH and after daily treatment over durations of up to and exceeding 1 month. Preclinical research and both positron emission tomography and single photon emission tomography of humans have shown that MPH increases extracellular dopamine and norepinephrine; the dose effects of MPH have an inverted *U*-shaped function where high doses may cause insomnia, nervousness, and increased heart rate among other symptoms and impair cognitive performance, whereas too low a dose fails to improve cognitive performance. In the past 5 years, small clinical trials, and experimental pilot studies have found therapeutic effects of single and repeated low doses of MPH in patients with mild TBI who reported cognitive dysfunction. This literature also suggests that MPH may interact with concurrent cognitive interventions to enhance their effects. This focused review will critically evaluate the recent literature on MPH effects on cognitive dysfunction after mild to moderate TBI. To elucidate the neural mechanisms of MPH effects, this review will also include recent imaging research, preclinical, and experimental human studies.

## Introduction

Methylphenidate (MPH) is a dopamine and noradrenaline agonist which has stimulant effects. It is widely prescribed in clinical settings (1) and is used in research. The primary objective of this review is to describe and critique clinical trials of MPH that have focused on improving cognitive performance and cognitive (“mental”) fatigue in persons who sustain mild to moderate traumatic brain injury (TBI). Although other catecholaminergic medications will be briefly considered, we will focus on MPH because it is the most investigated drug in this category, it is widely prescribed in rehabilitation, and in follow-up care for TBI (2, 3). Related objectives include examining the premise for using MPH to treat cognitive dysfunction in TBI; brain imaging and experimental evidence for the neural mechanisms which underpin MPH's effects; methodological issues in clinical trials of MPH; and its potential role as an adjuvant in cognitive rehabilitation. The clinical trials listed in Clinical trials.gov (see https://clinicaltrials.gov; Accessed February 14, 2019) that enrolled adult participants with a spectrum of TBI severity will be summarized. Finally, we will review the subset of published investigations that studied adult participants with mild to moderate TBI.

## Mechanism of Effects, Scientific Premise, and Inverted *U*-Shaped Function of Performance by Dose

### Mechanism of Therapeutic Effect on Cognition

The positive effects of MPH on cognition in conditions characterized by low dopamine are achieved by reducing reuptake of extracellular dopamine by the dopamine transporter (DAT) which is most densely represented in a group of contiguous subcortical structures in the forebrain, including the caudate, putamen, and nucleus accumbens and, to a lesser extent, in prefrontal cortex (3). Although MPH also affects reuptake of noradrenaline, the literature supports modulation of dopamine levels as the primary mechanism of clinical improvement in studies of TBI (3). MPH has also been the most frequently used dopamine agonist in clinical trials to treat cognitive deficit after TBI (3).

### Premise for MPH Treatment of Post-TBI Cognitive Deficit

The premise for using MPH to ameliorate cognitive deficits is based on several related lines of research. First, evidence suggests mediation of cognitive function by stimulation of dopaminergic and noradrenergic receptors in prefrontal cortex and in subcortical regions (3, 4). Additionally, the architecture of these neuromodulatory transmitter systems renders them vulnerable to injury as they originate within brainstem nuclei, project widely throughout the brain, and include neurons with long fibers, diffuse arborization, high baseline activity, and little or no myelination (3, 5). This diffuse distribution and fragile neuronal structures make these systems especially susceptible to both mechanical and metabolic injury (e.g., diffuse axonal injury) (3). Third, animal and clinical studies have provided evidence for catecholaminergic disruption following TBI (6, 7). Finally, dopamine levels have been shown to increase during the acute post-injury period within brain areas that include the medial prefrontal cortex, striatum, brainstem, and hypothalamus (3, 8). Such increases are then followed by a hypodopaminergic state characterized by a reduction in dopamine release and other alterations that decrease the overall level of dopaminergic function (3, 9).

Similarly, shortly after TBI there is also an acute increase in noradrenaline which is then followed by a reduction in noradrenergic activity (3). Imaging studies have confirmed these changes; positron emission tomographic imaging (PET) and single photon emission computed tomography (SPECT) have shown reduced dopamine transporter (DAT) binding secondary to lower dopamine levels (3, 4, 10). Jenkins et al. (10) also found that slow cognitive processing speed after moderate to severe TBI was specifically related to reduced DAT binding in the caudate. A recent translational SPECT investigation of moderate to severe TBI patients found that those with low pretreatment DAT level in the caudate showed significant improvement in complex reaction time (RT) after taking 0.3 mg/kg MPH for 2 weeks in a randomized cross-over design (11). In contrast, complex RT did not change in a placebo condition and MPH effects on performance were not significant in patients who had normal baseline levels of DAT. Although self-rated fatigue was reduced in both the low and normal baseline DAT level subgroups, a diminution of self-rated apathy was found only in the low DAT subgroup. In summary, these studies support the premise that MPH-related improvement in cognitive performance is mediated by increased dopamine level.

## Functional Magnetic Resonance Imaging (fMRI) Studies of MPH

### Application of Task Related fMRI to Study Neural Mechanisms of Cognitive Dysfunction After TBI

Task-related fMRI has been used to explore cognitive dysfunction following TBI, including studies employing pharmacological interventions and working memory paradigms [e.g., (12, 13)]. Following mild traumatic brain injury (mTBI), fMRI showed problems with the allocation of neural resources while engaged in working memory tasks. Activation of brain regions such as dorsolateral prefrontal cortex was excessive at low to moderate task difficulty levels, while higher task demands resulted in little or no additional increase in neural processing resources (12, 14). Preliminary research has started to address how modulation of brain activation might be improved through pharmacological manipulations (6). Although most fMRI studies involving pharmacologic agents have focused on moderate to severe TBI, we included them in Table 1 because they provide proof of principle concerning dopaminergic mechanisms in task-related activation and provide a framework for investigation of drug effects to treat persistent cognitive dysfunction after less severe TBI.

**Table 1:** MPH interventions in adults with a history of TBI ranging from mild to severe (see ClinicalTrials.gov).

**Study title and identifier**	**Brief description**	**Design and study population**	**Treatment schedule**	**Outcome measures**	**Study results and limitations**
Cognitive remediation after trauma exposure trial = CREATE Trial (CREATE) NCT01416948	To evaluate the efficacy of MPH and galantamine in the treatment of persistent cognitive symptoms associated with PTSD and/or TBI	Randomized, double-blind, placebo-controlled, parallel assignment; adults with mild to moderate TBI and/or PTSD	MPH 20 mg b.i.d., or galantamine 12 mg b.id., or placebo for 12 weeks	RNBI, RPSQ, RAVLT, TMT, subtests of WAIS-III, BVMT-R, PASAT, CPT, PTSD Checklist—specific event version, and Patient Health questionnaire-9	Study was terminated due to lack of recruitment-−32 participants out of proposed 159; Limitation- mixed TBI/PTSD population
Dopamine receptor imaging to predict response to stimulant therapy in chronic TBI NCT02225106	To evaluate PET imaging with [11C]-raclopride, a D2/D3 receptor ligand, before and after administering MPH, to measure endogenous dopamine release in TBI patients with problems in cognition, attention, and executive function	Non-randomized one-time placebo and one-time MPH, after that MPH for 4 weeks; adults with moderate to severe TBI	MPH 60 mg one-time, after that 30 mg b.i.d.; 4 weeks	CVLT, TMT, Subtests of the WAIS-IV, RPSQ, Sustained arousal and attention task 50/50; Dual task; Distraction task; Sustained attention to response, and Test of everyday attention	Study was completed with actual enrollment of 11 out of proposed 30; No results available; Limitations- small sample size, no randomization
MPH (Ritalin) and Memory/Attention in traumatic brain injury (TBI) NCT00453921	To compare the results of three interventions: memory and attention training, MPH, and memory/attention training in combination with MPH and use functional MRI to characterize changes in activation of the neural circuitry of memory and attention in study groups	Randomized, double-blind, placebo-controlled, parallel assignment; adults with mild to severe TBI	MPH 0.3 mg/kg b.i.d.; 7 weeks	CVLT, CPT, and Functional MRI task performance and brain activation (N-back)	All *p* < 0.05; Limitations- small sample size (18–20 participants in each group), wide range of TBI severity, and no info regarding participants' distribution of TBI severity
The relationship between traumatic brain injury and dopamine (a chemical in the brain) NCT02015949	To investigate if treatment with MPH improves cognitive functions in TBI, whether the mechanism involves a normalization of brain functioning and whether brain dopamine levels (measured by the SPECT and MRI) can predict the magnitude of any improvement in symptoms.	Randomized, cross-over, placebo controlled; adults ≥3 months post- moderate to severe TBI	MPH 0.3 mg/kg b.i.d. or placebo for 2 weeks	CRT and relationship of CRT to specific binding ratio of the dopamine transporter (DAT) in the striatum. Patients were divided into low vs. normal DAT level based on their DAT binding ratio on SPECT.	All participants completed trial (*n* = 40, 20 assigned to each MPH-placebo sequence). CRT was reduced (faster) in the low DAT subgroup while on MPH as compared to placebo; fatigue improved when on MPH.

*MPH, methylphenidate; TBI, traumatic brain injury; b.i.d., bis in die (latin) or two times a day; BVMT-R, Brief Visuospatial Memory Test-Revised; CPT, Continuous Performance Test; CRT, Choice Reaction Time task; CVLT, California Verbal Learning Test; MRI, Magnetic Resonance Imaging; RAVLT, Rey Auditory Verbal Learning Test; RNBI, Ruff Neurobehavioral Inventory—Post-morbid Cognitive Scale; RPSQ, Rivermead Post-concussion Symptom Questionnaire; PASAT, Paced Auditory Serial Addition Test; PTSD, post-traumatic stress disorder; SPECT, Single Photon Emission Computed Tomography; TMT, Trail Making Test; WAIS, Wechsler Adult Intelligence Scale*.

### Current Status of fMRI Studies Using Catecholaminergic Agents

MPH is thought to act through mechanisms that block the reuptake of dopamine and noradrenaline, as well as an increase in dopamine release (3). Together, such actions elevate extracellular concentrations of both of these catecholamines to produce stimulatory effects. However, the only pharmacological studies that have used functional neuroimaging to study mTBI have used other catecholaminergic agonists, bromocriptine and guanfacine, and their action differs from that of MPH (13, 15). Bromocriptine is a selective D_2_ dopamine receptor agonist with complex, dose-dependent effects (3). This agonist binds to presynaptic auto-receptors which inhibit dopamine release, as well as post-synaptic sites. At high doses, the excitatory post-synaptic effect is thought to predominate with a net result that facilitates dopaminergic function. In contrast, guanfacine is a selective α-2A adrenergic agonist which acts on receptors that are concentrated predominantly within the prefrontal cortex and the locus coeruleus, resulting in stimulation of the noradrenergic system (3, 6).

Using a single-dose pharmacological challenge approach with block design fMRI, Mcallister et al. (13) and Mcallister et al. (15) examined the effects of bromocriptine or guanfacine on activation during an auditory letter n-back working memory task. In one study, they administered guanfacine to 13 mTBI patients and 14 healthy control subjects within 1 month of injury as part of a double blind, placebo-controlled crossover design (15). The n-back task had three levels of difficulty and, for mTBI patients, noradrenergic stimulation improved performance at the intermediate level (i.e., 2-back), while the control subjects experienced a decline. Functional neuroimaging with the mTBI patients revealed an activation increase within the right frontal lobe (e.g., middle frontal gyrus) during the guanfacine condition, while the control subjects had activation increases within areas outside of working memory circuitry.

Mcallister et al. (15) used the same working memory paradigm and study design with 26 mTBI patients and 31 control subjects to investigate the D_2_ dopamine receptor agonist bromocriptine. This manipulation did not alter performance for the control subjects, but mTBI patients experienced declines during the 0-back and 3-back task conditions. The activation patterns found with bromocriptine were essentially the opposite of those observed with guanfacine, including increased frontal activation in the control subjects and increases outside of working memory circuitry for subjects with mTBI. When considered in combination, the findings for bromocriptine and guanfacine are consistent with different neural and behavioral responses to different types of catecholaminergic intervention after mTBI.

According to Mcallister et al. (6), alterations in central catecholaminergic sensitivity impair working memory and contribute to cognitive complaints shortly after mTBI, but the effectiveness of different pharmacological interventions for improving cognitive function likely depends upon factors such as the type of catecholaminergic stimulation and the dose. In particular, stimulation of the prefrontal noradrenergic system appears to have the potential to enhance working memory performance following mTBI (15). Although these studies (13, 15) did not examine MPH in patients with mTBI, others have used functional neuroimaging to study activation changes associated with MPH in patients with injuries of greater severity [e.g., (16)].

### Current Status of fMRI Studies Using MPH

Newsome et al. (16) examined the effects of a 1-month MPH intervention in patients with moderate to severe TBI. Using a double blind, placebo-controlled design, they administered either a placebo or 15 mg of the drug twice a day for a month with pre-treatment and end-of-treatment scanning performed using a block design visual n-back task with face stimuli. Four TBI patients received MPH and the other five were in the placebo group. In a whole brain analysis examining the 2-back minus 0-back contrast, this MPH treatment, relative to placebo, reduced activation within areas thought to have a role in working memory, such as the anterior cingulate gyrus, cuneus, and cerebellum. An *a priori* region of interest analysis also found treatment-related reductions within the anterior cingulate gyrus.

Studies of moderate to severe TBI using single-dose pharmacological challenge approaches have also provided evidence for alterations in working memory activation following MPH administration (17, 18). Manktelow et al. (18) used a double blind, placebo-controlled crossover design to study the effects of a single 30 mg. dose in 15 patients with moderate to severe TBI and 15 healthy controls. Using a block design visual letters n-back task, Manktelow et al. reported that the controls performed better than the TBI patients during the placebo condition, but there was no significant between-group difference after administration of the MPH. In TBI patients the drug increased task-related activation within a portion of the left cerebellum to a level comparable to controls and this change was correlated with the improvement in working memory performance. Kim et al. (17) used perfusion fMRI and a block design visual letters n-back task with 21 moderate to severe TBI patients. The pharmacological challenge consisted of a single dose (0.3 mg/kg) of MPH that was delivered as part of a randomized double-blind, placebo-controlled crossover study design. The MPH improved RT, with a trend toward greater task accuracy, and on functional neuroimaging there was also a trend toward a global reduction of cerebral blood flow under all of the task conditions, including the rest blocks. These findings suggest the possibility of a general mechanism of action for cognitive enhancement associated with MPH in patients with moderate to severe TBI.

Visual attention and response inhibition are cognitive functions that have also been studied in moderate to severe TBI patients using MPH (17, 19). In addition to the n-back working memory paradigm that was described above, Kim et al. (17) also employed the Visual Sustained Attention Task (VSAT) block design fMRI paradigm with 18 of their study participants. The administration of a single dose of MPH (0.3 mg/kg) improved both VSAT RT and accuracy. Also, during the MPH condition, there was deactivation within the left posterior superior parietal cortex that was correlated with improved RT. These authors concluded that suppression of activation within this brain region may represent a mechanism through which MPH improves visual attention impairment following TBI. Use of a single 30 mg dose has also been found to be related to activation on event-related fMRI using the stop signal task, which is a challenging measure of response inhibition. Moreno-López et al. (19) used this fMRI paradigm in a randomized double blind, placebo-controlled crossover study with 14 moderate to severe TBI patients and 20 healthy controls. Under the placebo condition the TBI patients had decreased task-related activation, relative to control subjects, within the right inferior frontal gyrus. However, the administration of MPH increased activation within this structure to a level that was similar to that of the control subjects.

**Figure 1:**
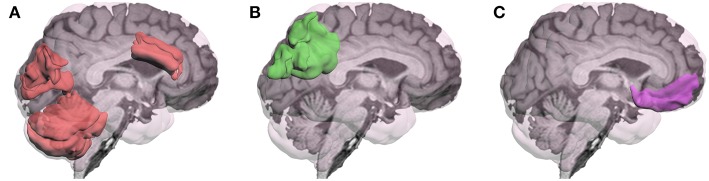
Brain regions in which methylphenidate (MPH) has modulated activation during performance of cognitive tasks in patients with TBI have varied depending on the specific cognitive task: **(A)**Visual Working Memory: activation by an n-back task for photos of faces was modulated in anterior cingulate gyrus, cuneus, and cerebellum (16), and for letters in left cerebellum (18); **(B)** Visual Sustained Reaction Time (RT): MPH modulated deactivation of left posterior superior parietal region (17); **(C)** Response Inhibition: MPH modulated activation by the stop signal RT task in the right inferior frontal gyrus (19).

In summary, fMRI research addressing neural mechanisms associated with MPH's cognitive effects following TBI has been limited. Although previous fMRI research has often reported the presence of greater and more diffuse activation following TBI (20–22), the more recent task-related fMRI studies of MPH found that the drug altered activation in the direction approximating healthy controls. However, the brain regions most affected by MPH depended on the specific tasks used as represented in Figure 1. These changes in activation were generally correlated with improved cognitive performance.

These findings were interpreted as providing evidence for the normalization of working memory activation following the 1-month MPH intervention. To our knowledge, no studies have specifically studied activation changes in patients with mTBI using MPH, but the other catecholaminergic drugs guanfacine and bromocriptine have been examined using a working memory paradigm, and the findings suggest the possibility of different effects associated with the stimulation of dopaminergic and noradrenergic systems (6, 13, 15). Since MPH acts on both of these neuromodulatory systems (i.e., MPH is a dual agonist), there is a justification for further research to determine how this particular drug alters brain function to improve performance following mTBI. However, several studies with moderate to severe TBI have been conducted and, although the details of their findings are inconsistent, there is some preliminary evidence that administration of MPH may normalize the pattern of activation in a way that enhances performance.

High variability in the cognitive response and functional imaging findings may reflect heterogeneity of the neuropathology associated with TBI (3). Current status of fMRI studies using individual differences in the level and type of injury-related cognitive impairment (3, 6), non-linear relationships between catecholamine levels and cognitive function (23), subject factors such as age and genetics (3, 6), and between-study differences in research design and statistical power, also contribute to the variability in findings across studies (22). Differences in the reduction of dopamine among patients sustaining TBI of apparently similar acute severity is another source of variability in response to MPH both in changes of task related activation and improvement in cognitive performance.

## Clinical Trials of MPH and Other Catecholaminergic Drugs

Clinical trials of MPH have studied neurologic disorders involving low levels of brain dopamine, especially in the striatum. Currently over 300 clinical trials of MPH have been posted on the ClinicalTrials.gov website with 195 of them on adults (see https://clinicaltrials.gov/ct2/results?term=methylphenidate&cond=brain+injury, accessed February 01, 2019). The majority of these studies involve developmental attention-deficit/hyperactivity disorder (ADHD), followed by narcolepsy and chronic fatigue syndrome due to multiple sclerosis, other autoimmune conditions, or as a result of cancer treatment. Several trials of MPH were designed to enhance gait and balance in Parkinson's disease and to alleviate apathy in patients with Alzheimer's disease or other dementia. In contrast, relatively few trials of MPH were designed to improve cognitive functioning following TBI.

Clinical trials of MPH to treat cognitive deficits after TBI have mostly enrolled moderate to severely injured patients and these studies have been reviewed recently (2, 3, 10). Table 1 summarizes those trials in Clinical Trials.gov that focused on moderate to severe TBI or enrolled patients representing a spectrum of TBI severity. Table 2 summarizes the clinical trials of MPH and related drugs that have enrolled mild to moderate TBI patients. These clinical trials to date have aimed at treating cognitive deficit rather than impacting disability. The clinical trials included in Table 2 were identified by searching PubMed, published meta-analyses, and reviews.

**Table 2:** Clinical Trials of Methylphenidate in Adults with History of Mild to Moderate TBI.

**Article (First author and year)**	**Participants in each group (N), mean age (SD), Chronicity**	**Study design, max MPH dose, duration of treatment**	**Cognitive measures**	**Results/ *p-*values**	**Limitations**
Lee et al. (24)	MPH (*N* = 10); 35.3 (8.0) Placebo (*N* = 10); 35.5 (7.2); Chronicity: 2weeks-1 year;	Randomized, double-blind, placebo-controlled, parallel design; 20 mg; 4 weeks	Critical flicker fusion threshold, CRT, CTT, MAT, Sternberg memory scanning task, DSST, and MMSE	Significant treatment effect *p* < 0.05	All participants were diagnosed with major depression; small sample size
Johansson et al. (25)	*N* = 29; 38.6 (11.1); Chronicity:8.6 years (5.1)	Prospective, open-label, crossover; No MPH, Low dose MPH, Normal dose MPH- 4 weeks/each; max dose- 20 mg	MFS	Significant treatment effect with *p* < 0.001	No placebo-control; small sample; no cognitive testing; participants Selected or mental fatigue and pain ≥ 12m
Johansson et al. (26)	*N* = 44; 38.9 (10.8); Chronicity: 8.2 years (5.7)	Prospective, open-label, crossover; No MPH, Low dose MPH, Normal dose MPH- 4 weeks/each; max dose- 20 mg	DSC and DS (WAIS-III), TMT, and MFS	DSC: *p* = 0.04 MFS: *p* < 0.001 All other: *p* > 0.1	Lack of placebo-control; patients selected for moderate disability, mental fatigue, pain
Mcallister et al. (27)	MPH (*N* = 9); 36.7 (9.3) Placebo (*N* = 12): 44.4 (8.2) Chronicity: N/A	Randomized, double-blind, placebo-controlled; 20 mg; 12 weeks	RAVLT, DS, RNBI, RPSQ, and TMT	RNBI: *p* = 0.004 DS: *p* = 0.011 All other: unknown	Small sample size; mixed mTBI/PTSD population in both groups
Johansson et al. (28)	*N* = 30; 39.7 (12.5); Chronicity: 8.6 years (5.9)	Prospective, open-label, max dose- 20 mg; 6 months; patients were responders to MPH in prior phase	DSC and DS (WAIS-III), TMT, and MFS	All *p* < 0.001	Lack of placebo-control; small sample size;
Zhang and Wang (29)	MPH (*N* = 18); 36.3 (10.9); Placebo (*N* = 18): 34.9 (12.1); Chronicity: 46.5 days (6.8), MPH; 46.1 days (7.2), placebo	Randomized, double-blind, placebo-controlled; 30 weeks	MFS, CRT, CTT, MAT, DSST, and MMSE	MFS: *p* = 0.005 MAT: *p* = 0.02 All other: *p* < 0.001	Small sample size
Jonasson et al. (30)	*N* = 18; 44.9 (10.4); Chronicity: ~22 months post-injury; patients were responders in prior trial	Prospective, open-label, max dose- individual; 4 weeks after a 4 weeks period off MPH	DSC and DS (WAIS-III), and MFS	DSC: *p* < 0.001 DS: *p* = 0.011 MFS: *p* < 0.001	Lack of placebo-control design; small sample size

*MPH, Methylphenidate; TBI, traumatic brain injury; CRT, Choice Reaction Time task; CTT, Compensatory Tracking Task; DS, Digit Span; DSC, Digit Symbol Coding; DSST, Digit Symbol Substitution Test; MAT, Mental Arithmetic Test; MFS, Mental Fatigue Scale; MMSE, Mini-Mental State Examination; N/A, not applicable; RNBI, Ruff Neurobehavioral Inventory-Post-morbid Cognitive Scale; RPSQ, Rivermead Post-concussion Symptom Questionnaire*.

The trials described below used MPH to treat persistent cognitive symptoms, cognitive impairment, and cognitive (or “mental”) fatigue following mild to moderate TBI, including patients who had mild or no impairment of consciousness but sustained brain lesions or other pathology identified by imaging (“complicated mTBI”). The rationale for focusing on mild to moderate TBI is that this range of severity accounts for over 80% of the 2.8 million acute TBI population treated in emergency departments annually in the USA (31). The subgroup of the mild to moderate TBI population who have cognitive impairment persisting for 3 months or longer is estimated to be ~15–30% which represents a large, underserved population (32). However, there is a paucity of high-quality longitudinal follow-up studies using cognitive tests to evaluate recovery in patients with this range of acute TBI severity. This gap in clinical trials of MPH for cognitive deficit after mild to moderate TBI is concerning because cognitive dysfunction impacts return to work and other activities which affect quality of life. In addressing this gap in clinical trials of MPH, it is important to consider the methodological issues in study design and conduct as described below.

## Materials and Methods

### Methodological Issues in MPH Clinical Trials

#### Variation in Severity and Chronicity of TBI

There is wide variation in the severity and chronicity of TBI represented in trials that have enrolled a spectrum of TBI severity (Table 1). However, the studies summarized in Table 2 are more homogeneous as they enrolled patients with mild to moderate TBI.

#### Eligibility Criteria for Enrollment

There is also variation across trials in the eligibility criteria for enrollment; some screened for impaired cognitive performance in addition to self-report of cognitive dysfunction, whereas other studies relied on self-report, clinical observations and clinical judgment, and/or report by a collateral source. Studies have also differed in screening for co-morbidities, including depression, anxiety, post-traumatic stress disorder and ADHD; some trials excluded depressed patients to isolate cognitive effects of MPH from MPH- related improvement of mood. Screening for symptom validity and effort is also an issue because patients seeking compensation may exaggerate their cognitive impairment or expend less than full effort in their performance on cognitive tests.

#### Study Design

Table 2 shows variation in study design; randomized, clinical trials using placebo-control groups have been limited by small sample sizes, whereas crossover designs have mitigated this problem. An additional advantage of crossover designs is that they are robust to the considerable heterogeneity in TBI pathology even in patients with equivalent TBI severity. The short washout (≈24 h) of MPH is also well-suited for crossover designs as placebo and drug conditions can be scheduled with separation by a brief interval. Administering MPH at the same time each day is also recommended to mitigate confounding by chronobiologic variation. However, practice effects on cognitive tests are a potential confound, arguably more so in crossover designs, especially those that are open label. In addition, patients may experience increased arousal which cues them to the MPH phase of the study.

#### MPH Dose and Duration

Studies have ranged in duration of treatment from single administration of MPH and same day “challenge” testing to the cohort followed by Johansson et al. (30) who maintained MPH responders for 2 years following a 4-months interval without treatment. Table 2 shows that the dose of MPH has ranged from 20 to 30 mg in studies using a fixed dose; other studies have used 0.3 mg/kg with the constraint of a maximum dose. Johansson et al. (30) have used an individualized dose escalation strategy which is atypical in the literature. Johansson et al. (30) maintained MPH responders for 2 years following a 4-months interval without treatment. She reported that MPH effects dissipated during this 4-months drug-free period, but the therapeutic effects of MPH on processing speed, working memory, and cognitive fatigue were reinstated when the patients resumed MPH according to the regimen described below.

In a crossover trial to treat cognitive fatigue, Johansson et al. (30) used a Latin Square design wherein each patient had 4 weeks in each of three different conditions: (1) no medication, (2) low dose MPH, and (3) normal dose MPH. There was no washout period because a short-acting preparation of MPH was used. The low and normal dose conditions included dose escalation during weeks 1–3, which rose to 60 mg/day in the fourth week of the normal dose condition. Of 29 patients (age 18–65 years) who were enrolled in the trial, 5 dropped out including 4 subjects who reported adverse events. Reduction of symptoms as measured by the Mental Fatigue Scale (MFS) (33), was greater in the normal and low dose MPH conditions as compared to no medication and symptom reduction under the normal dose exceeded that of the low dose condition. Conceptually, the MFS has ecological relevance because it queries about variation in fatigue at different times of the day and its effects on psychological health and sleep. In this respect, the MFS compliments measures of cognitive performance which may take an hour or two but possibly overlook the cumulative effects of work or study over hours in the course of the patient's daily schedule (33).

**Figure 2:**
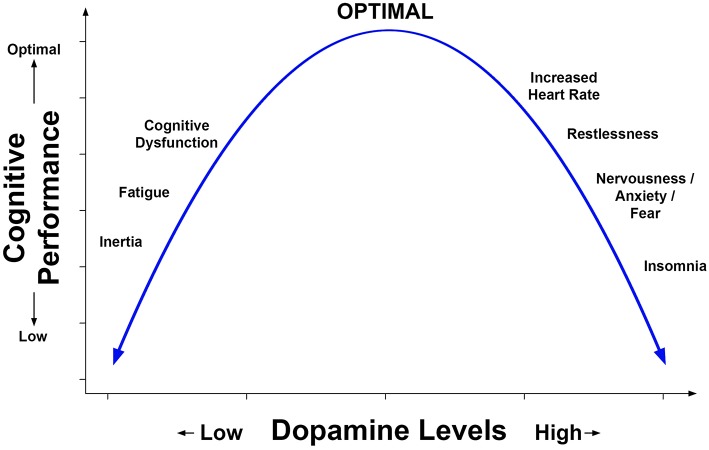
Representation of the inverted *U*-shaped relation of prefrontal dopamine level to cognitive performance and symptoms. This relation is based in part on animal models including the work by Arnsten et al. (4) and has been described in reports concerning the effects of dopamine agonists on cognition and behavior in humans.

Although dose escalation adds complexity to a trial, it may be more representative of clinical practice and is advisable for older patients. To this point, Jonasson et al. (30) screened for cardiovascular health, including electrocardiography at each visit. The Jonasson et al. study's dose escalation approach is noteworthy because it may have optimized MPH treatment, ostensibly reaching the top of the inverted *U*-shaped function as represented in Figure 2.

#### Inverted U-Shaped Relation of Dose Level to Performance and Adverse Effects of MPH

The results of experimental research using animal models and clinical studies are consistent with an inverted U-shaped relation of cognitive performance to dose of MPH. Figure 2 is a hypothetical representation of the results showing that cognitive performance is optimized by a moderate level of dopamine. This relation has been inferred (but not proven) from dose ranging studies of MPH which lacked a measure of dopamine. Suboptimal levels of brain dopamine are associated with fatigue, whereas excessively high levels can increase heart rate and produce symptoms such as nervousness, increased motor activity, and sleep disturbance. Baseline levels of dopamine are lower in older adults and reduced in neurologic conditions such as Parkinson's disease. A recent clinical SPECT investigation indirectly measured dopamine level by evaluating the binding of a radio ligand to DAT which was especially evident in the caudate (10). Consistent with the inverted *U*-shaped relation, these investigators found that moderate to severe TBI patients responded better to MPH on cognitive tests and reduction of fatigue if their baseline levels of brain dopamine were low, whereas patients who had normal levels of dopamine did not respond as well. Based on the above studies, screening for medical history and substance abuse, serial recording of adverse effects reported by the participant, and repeated measurement of vital signs is good practice for clinical trials of MPH.

#### Outcome Measures

Similar to variation in the enrollment criteria, clinical trials have varied in their use of self-report vs. cognitive performance measures. As described in the preceding section, cognitive (“mental”) fatigue is a frequent complaint in the TBI population wherein the individual may be capable of performing the cognitive demands of a task or activity, but finds the process to be effortful and tiring as compared to her/his pre-injury level. Patients also reported that cognitive fatigue was present throughout the day. Interestingly, rating scale and visual analog scale measures of fatigue have been sensitive to MPH effects (30). In a recent translational study in moderate to severe TBI patients, Jenkins et al. (10) found that cognitive fatigue was sensitive to MPH in subgroups of participants who differed in level of pretreatment dopamine in the caudate based on SPECT using a ligand for dopamine.

Of the cognitive performance measures, complex RT, go no-go RT, cognitive processing speed, and set shifting tests have been widely used. Episodic multi-trial recall memory and working memory tests have also been employed, but less frequently than processing speed and RT tests. Tables 1, 2 show that some studies have relied on self-report of cognitive functioning in everyday activities as measured by various scales. From the perspective of ecological validity, a combination of cognitive performance and self-report measures is recommended as used by Jenkins et al. (10).

Few clinical trials of MPH have used composite measures to assess cognitive performance. Although a composite measure has the advantage of evaluating diverse cognitive operations, specific cognitive tasks such as working memory are supported by preclinical research implicating prefrontal dopamine receptors (4) and measures of cognitive processing speed have been especially sensitive to MPH in clinical trials. As seen in Table 2, timed tests that have been sensitive to MPH include Trail Making, Complex RT, Digit Symbol Substitution and Coding subtests, and Sternberg Memory Scanning, which measures changes in RT depending on the number of items to be held in memory.

#### Concurrent Cognitive Training and MPH Treatment

In view of the positive effects of MPH on attention, processing speed, and fatigue, it is plausible that MPH may enhance the effects of cognitive training. In a study which enrolled patients representing a wide spectrum of acute TBI severity, Mcdonald et al. (34) found that 0.3 mg/kg of MPH taken over 7 weeks enhanced the effects of memory and attention training in a controlled study.

## Discussion

Despite relatively few postings on ClinicalTrials.gov, there is a considerable body of literature related to using MPH to treat cognitive complaints, cognitive deficits, and mental fatigue following mild to moderate TBI (2). Most of the previous reviews included studies with a wide range of TBI severity (with majority of them on moderate to severe range) (35, 36) and age (from children to older adults) (37). All of the above-mentioned studies have considerable limitations due to small sample sizes, enrollment of individuals with co-morbid depression (24, 27) or use of open-label design (25, 26, 28, 30). Hence, there is an evident need for well-designed and adequately powered, double blind, placebo-controlled clinical trials that will extend our knowledge of neural mechanisms of MPH effects and provide valuable information for clinicians and researchers.

Of considerable note, there is no consensus on whether to include screening measures of cognitive performance to substantiate self-report of cognitive dysfunction in everyday activities. If cognitive performance measures are used to screen for eligibility, they should tap the constructs that patients complain about. Few studies have obtained ratings of cognitive function by collateral sources and measures of symptom validity or effort expended during testing have generally not been used. Based on Mcdonald et al.'s (34) work, trials combining MPH with cognitive training also appear to be justified. Imaging biomarkers of dopamine and/or MPH effects also enhance the rigor of clinical trials.

## Conclusions

The extant evidence supports further investigation of MPH for use in treating cognitive dysfunction and mental fatigue following mild to moderate TBI. However, there is a need for phase 3 clinical trials to evaluate the effectiveness of MPH and identifying the specific context of use in which it is most strongly indicated.

## Author Contributions

HL, RS, and MT wrote the main manuscript text. EW, TA, and JP edited the paper. TA designed and produced the figures. JH reviewed and verified manuscript and figures. JP revised and formatted the manuscript according to the journal's guidelines.

### Conflict of Interest Statement

The authors declare that the research was conducted in the absence of any commercial or financial relationships that could be construed as a potential conflict of interest.
